# Role of left atrial appendage occlusion in patients with HeartMate 3

**DOI:** 10.1093/icvts/ivab285

**Published:** 2021-10-18

**Authors:** Andrew Melehy, Gillian O’Connell, Yuming Ning, Paul Kurlansky, Yuji Kaku, Veli Topkara, Melana Yuzefpolskaya, Paolo C Colombo, Gabriel Sayer, Nir Uriel, Yoshifumi Naka, Koji Takeda

**Affiliations:** 1 Department of Surgery, Division of Cardiothoracic and Vascular Surgery, Columbia University Medical Center, New York, NY, USA; 2 Department of Surgery, Center of Innovation and Outcomes Research, Columbia University Medical Center, New York, NY, USA; 3 Department of Medicine, Division of Cardiology, Columbia University Medical Center, New York, NY, USA

**Keywords:** Left atrial appendage occlusion, HeartMate 3, Mechanical circulatory support, Thromboembolism

## Abstract

**OBJECTIVES:**

Left atrial appendage occlusion (LAAO) at the time of implantation may reduce thromboembolic events (TEs) during continuous-flow left ventricular assist device support. The HeartMate 3 (HM3) reduces TEs overall, but the efficacy of LAAO in HM3 is unknown.

**METHODS:**

Adults receiving first HM3 implantation from November 2014 through December 2019 at a single, large medical centre were retrospectively reviewed. TEs included device thrombosis and ischaemic stroke. Patients were classified by whether they received LAAO or not. Incidence of TEs was compared between groups using cumulative incidence curves with competing risks (death and heart transplant) and risk factors for TEs were assessed with Fine and Gray competing risk regression.

**RESULTS:**

A total of 182 patients received HM3, of whom 99 (54%) received LAAO versus 83 (46%) who did not. There were 14 TEs, including 13 strokes (7%) and 1 pump thrombosis (0.5%). No significant difference in the incidence of TEs in each group was found (Gray’s test: *P* = 0.35). LAAO was not associated with TEs in multivariable Fine–Gray analysis (*P* = 0.10) and no significant risk factors for TEs were found. There were zero disabling strokes in those who received LAAO compared to 6 (7%) in those who did not receive LAAO (*P* = 0.008).

**CONCLUSIONS:**

A low number of TEs was observed in HM3 recipients. LAAO did not further reduce the overall rate of TEs in this patient population, though its use may be beneficial in preventing disabling ischaemic strokes after HM3 implantation.

**Figure:**
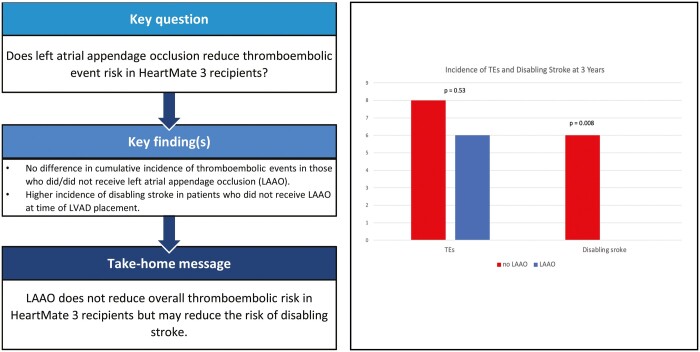

## INTRODUCTION

Thromboembolic events (TEs) such as ischaemic stroke and device thrombosis have historically been significant complications associated with non-HeartMate 3 (HM3) continuous-flow left ventricular assist devices (LVADs) [1–4]. Reported risk factors for ischaemic stroke in LVAD patients include atrial fibrillation, female sex and hypertension [1, 2, 5]. Increased CHA_2_DS_2_–VASc scores have also been associated with TEs after LVAD implantation [6]. Left atrial appendage occlusion (LAAO) is associated with a decreased risk of stroke in patients with atrial fibrillation [7, 8], and concomitant LAAO at the time of LVAD placement increases TE-free survival relative to those who did not receive LAAO [9].

The HM3 is the newest generation of continuous-flow LVADs that utilizes a centrifugal pump system and fully magnetically levitated component parts [10]. The HM3 is associated with significantly improved TE-free survival at 2 years compared to previous generations of continuous-flow LVADs [10]. The HM3 is also associated with a dramatic reduction in pump thrombosis as well as reductions in both total and disabling ischaemic strokes at 2 years post-implantation [10]. The disabling ischaemic stroke rate between HM3 and previous continuous-flow LVADs is similar in the first 6 months after implantation, with the reduction in disabling ischaemic stroke risk primarily occurring after 6 months post-implantation [11]. Factors associated with survival free of haemocompatibility-related adverse events (TEs, neurological events, bleeding) after HM3 implantation include older age as well as international normalized ratio <1.5 and not being on aspirin at 30 days postoperatively [11]. LAAO was not associated with haemocompatibility-related adverse events following HM3 implantation. Prior studies on haemocompatibility-related adverse events in HM3 patients have included a low number of patients with LAAO in analyses and included all causes of stroke, including bleeding events, in the outcomes of interest. This study examines the effect of LAAO concomitant with HM3 implantation on TE risk specifically to determine if LAAO at the time of HM3 implantation reduces TEs.

## PATIENTS AND METHODS

### Ethical statement

This study was approved by the Columbia University Medical Center Institutional Review Board Protocol AAAE1866 with the waiver of informed consent.

### Study design

Patients who received a first HM3 implant at a single, large medical centre from November 2014 through December 2019 were retrospectively reviewed. Primary and secondary end points of interest were the incidence of TEs and disabling stroke, respectively.

### Data collection

All data were collected from the electronic medical record. All LVAD recipients included in this study were enrolled consecutively. Recorded data included baseline demographics such as past medical histories, INTERMACS score, preoperative laboratory data and use of an additional mechanical circulatory support tool prior to LVAD implant such as an extracorporeal membrane oxygenation, Impella or intra-aortic balloon pump. Haemodynamic data prior to implant including central venous pressure, mean pulmonary artery pressure, pulmonary capillary wedge pressure and Fick cardiac output were also collected. Operative characteristics including whether patients received LAAO or not and outcomes data including short- and long-term outcomes and TEs were collected. TEs included ischaemic stroke and device thrombosis. Consistent with the American Heart Association, in this study, stroke is defined as clinical symptoms attributable to ischaemia or transient ischaemic attack and excludes patients with the evidence of cerebral haemorrhage based on imaging [12]. Given this, and this study’s primary end point of TEs, patients with imaging indicative of haemorrhagic stroke were excluded from this study. Disabling stroke was defined as persistent clinical neurological deficit attributable to ischaemic stroke that required further therapy or resulted in coma or brain death. Patients were monitored for TEs via routine clinical follow-up. Short-term outcomes included in-hospital mortality and morbidity and postoperative atrial fibrillation. In-hospital morbidities included sepsis, urinary tract infection, takeback, use of renal replacement therapy and tracheostomy. Postoperative atrial fibrillation was defined as any atrial fibrillation occurring within 30 days of HM3 implantation regardless of the past history of atrial fibrillation.

### Operative use of LAAO

During this study period, the standard approach for HM3 implantation was a full median sternotomy. Patients’ initial LVAD settings were determined intraoperatively by assessing haemodynamics, septal shift, degree of mitral regurgitation and size of aortic valve opening. LAAO was performed at the surgeon’s discretion but was generally indicated in all patients for TE prevention, regardless of atrial fibrillation history or prior stroke. All LAAOs were performed with the AtriClip LAA Exclusion System (AtriCure, Inc, Mason, OH, USA). The main obstacle to performing LAAO at the time of HM3 implantation was the difficulty in accessing the left atrial appendage from outside. Therefore, patients with repeat sternotomy were not generally candidates for concomitant LAAO. Complete closure and lack of residual communication between the left atrium and appendage was confirmed by external inspection and transoesophageal echocardiography.

### Postoperative anticoagulation protocol

All patients were treated with antiplatelet therapy and warfarin with an international normalized ratio goal of 2–3. International normalized ratio goals were adjusted on an individual patient basis; a lower international normalized ratio was targeted for patients with bleeding events and patients with thrombotic events received therapeutic anticoagulation.

### Follow-up

Patients were followed via routine clinical follow-up from the time of LVAD implantation to cardiac transplant, death or the end of the review period (31 January 2020). All patients were monitored via an outpatient LVAD clinic at this institution.

### Statistical analysis

All analyses were conducted using R. *P*-values <0.05 were considered significant. Continuous variables were assessed for normality (Shapiro–Wilk test) and reported as median (interquartile range) or mean (standard deviation). Normally distributed data were compared using Student’s *t*-test and non-normally distributed data were compared using the Mann–Whitney *U*-test. Categorical variables were compared using Chi-square or Fisher’s exact test (any group having fewer than 10 observations). To compare the incidence of TEs between those who received LAAO and those who did not, cumulative incidence curves were created with death and heart transplant as competing risks. Predictors of TEs were investigated with Fine and Gray competing risk regression. Variables that were found to be significant in the univariable Fine and Gray regression (*P* < 0.05) were included in a multivariable analysis with LAAO. The incidence of disabling strokes between those who received LAAO and those who did not was compared using the chi-square test. Cumulative incidence curves and Fine and Gray regression could not be used to compare disabling strokes between groups as the LAAO group had zero disabling strokes.

Survival was compared between groups via Cox proportional hazards analysis in which patients were censored at the time of transplant or loss to follow-up. Variables found to be significantly associated with survival (*P* < 0.05) in univariable Cox models were included in a multivariable model with LAAO. Adjusted survival curves comparing survival in those who received LAAO and those who did not (adjusting for covariates significantly associated with mortality) were created. The effect of LAAO on in-hospital mortality was assessed via univariable logistic regression. A multi-variable regression could not be performed due to the low number of in-hospital mortalities. Variables with 5% or more data missing were excluded from all regression analyses, which included the history of peripheral vascular disease, discharge international normalized ratio, discharge haemoglobin and cardiopulmonary bypass time. Listwise deletion was utilized for variables with any missing data for regression analyses.

## RESULTS

### Patient characteristics

Of the 182 patients who received HM3 implants, 99 (54%) received concomitant LAAO. Those who received LAAO tended to be younger (median age 59 vs. 66 years, *P* < 0.001). The median CHA_2_DS_2_-VASc score was 3 in both groups (no LAAO IQR 3–4, LAAO IQR 2–4, *P* = 0.017). The LAAO group had a lower percentage of patients with a history of ischaemic cardiomyopathy (26% vs 63% in those without LAAO, *P* < 0.001). Four patients (4%) in the LAAO group had a previous sternotomy, compared to 43 (52%) in the no LAAO group (*P* < 0.001). Complete demographic comparisons between those who received LAAO and those who did not are shown in Table 1. Five patients had intracardiac thrombus discovered at the time of LVAD implant (2 in the no LAAO group vs 3 in the LAAO group, *P* = 1.00). Four intracardiac thrombi were discovered in the left ventricle intraoperatively and 1 in the left atrial appendage.

**Table 1:** Baseline demographics and operative characteristics comparing patients who did and did not receive left atrial appendage occlusion

Variable	No LAAO (*n* = 83)	LAAO (*n* = 99)	*P*-value
Baseline demographics			
Age, years	66 (56.03–70.37)	59 (50.79–63.14)	<0.001
Sex, male	88 (73)	80 (79)	0.20
HTN	66 (55)	60 (59)	0.44
Stroke/TIA	16 (13)	17 (16)	0.97
PVD	11 (9)	6 (5)	0.26
COPD	10 (8)	12 (12)	0.77
DM	46 (38)	35 (35)	0.20
Afib	55 (45)	48 (46)	0.48
ICM	63 (52)	26 (26)	<0.001
Previous sternotomy	52 (43)	4 (4)	<0.001
Previous tricuspid surgery	1 (1)	2 (2)	1.00
Previous mitral surgery	8 (7)	5 (5)	0.55
Prior CABG	42 (35)	0 (0)	<0.001
BTT	25 (21)	14 (14)	0.087
CHA_2_DS_2_-VASc	3 (3–4)	3 (2–4)	0.017
INTERMACS			0.71
1	14 (12)	11 (11)	
2	46 (38)	54 (53)	
3	33 (27)	30 (30)	
4	7 (6)	5 (5)	
IABP	31 (26)	45 (44)	0.076
Impella	6 (5)	1 (1)	0.094
ECMO	6 (5)	8 (8)	0.77
mPAP, mmHg	33.95 ± 10.62	35.52 ± 9.87	0.30
PCWP, mmHg	22.55 ± 9.15	23.85 ± 9.24	0.35
CVP, mmHg	10 (6–14)	9 (6–15)	0.96
Fick cardiac output, l/min	3.58 (2.94–4.46)	3.59 (2.88–4.19)	0.75
Preoperative creatinine, mg/dl	1.46 ± 0.45	1.4 ± 0.41	0.36
Preoperative albumin, g/dl	3.8 (3.2–4.15)	3.8 (3.32–4.18)	0.48
Operative characteristics			
CPB time, min	103 (83–125)	96.2 (72.8–132.5)	0.52
Concomitant surgery			
Aortic valve surgery	19 (16)	15 (15)	0.59
Mitral valve surgery	10 (8)	19 (19)	0.094
Tricuspid valve surgery	8 (7)	7 (7)	0.78
Intraoperative intracardiac thrombus	2 (2)	3 (3)	1.00
Left ventricle	2	2	
Left atrium	0	0	
Left atrial appendage	0	1	

Data are presented as % (*n*) for categorical variables and median (interquartile range) or mean ± standard deviation for continuous variables.

Afib: atrial fibrillation; BTT: bridge to transplantation; CABG: coronary artery bypass grafting; COPD: chronic obstructive pulmonary disease; CPB: cardiopulmonary bypass; DM: diabetes mellitus; ECMO: extracorporeal membrane oxygenation; HTN: hypertension; IABP: intra-aortic balloon pump; ICM: ischaemic cardiomyopathy; LAAO: left atrial appendage occlusion; mPAP: mean pulmonary artery pressure; PCWP: pulmonary capillary wedge pressure; PVD: peripheral vascular disease.

#### Early and late outcomes

**Figure 1:**
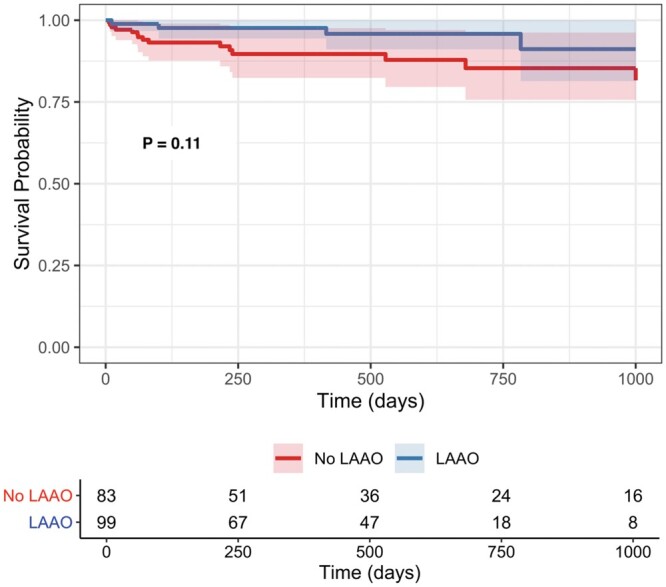
Adjusted survival curves comparing survival in patients who received left atrial appendage occlusion with HeartMate 3 implant and those who did not based on a multivariable Cox analysis including left atrial appendage occlusion, age and previous sternotomy. *P*-value is from left atrial appendage occlusion in the multivariable Cox model. LAAO: left atrial appendage occlusion.

Data related to mortality and morbidity are shown in Table 2. The median time to event or last follow-up was similar between groups (422 days in the no LAAO group vs 461 in the LAAO group, *P* = 0.93). Those who received LAAO had similar incidences of postoperative morbidities compared to the no LAAO group, including similar incidences of postoperative atrial fibrillation (35% vs 41%, *P* = 0.46). In-hospital mortality was lower in the LAAO group (odds ratio: 0.92, 95% confidence interval [0.85–0.98], *P* = 0.013). Survival was not significantly different between the LAAO and no LAAO groups as assessed by multivariable Cox proportional hazards analysis (hazard ratio: 0.39, 95% confidence interval [0.12–1.23], *P* = 0.11; Fig. 1). Complete Cox proportional hazards data is shown in Table 3. There were no identified risk factors for mortality in multivariable analysis.

**Table 2:** Outcomes Data comparing patients who did and did not receive left atrial appendage occlusion

Variable	No LAAO (*n* = 83)	LAAO (*n* = 99)	*P*-value
Postoperative atrial fibrillation	35 (29)	41 (41)	0.46
Postoperative VT/VF	20 (16)	22 (22)	0.77
Postoperative sepsis	16 (13)	19 (19)	0.72
Postoperative UTI	15 (12)	21 (21)	0.34
Postoperative takeback	12 (10)	17 (17)	0.47
Postoperative RRT	9 (7)	4 (4)	0.23
Postoperative tracheostomy	10 (8)	9 (9)	1.00
Thromboembolic events	10 (8)	6 (6)	0.53
Ischaemic strokes	10 (8)	5 (5)	0.26
Time to ischaemic stroke, days	7 (3.25–23.25)	67 (12–123)	0.093
Disabling strokes	7 (6)	0 (0)	0.008
Cardiac transplants	24 (20)	18 (18)	0.43
Hospital stay, days	32 (20.460–49)	27.5 (21–36.25)	0.25
Overall mortality	18 (15)	5 (5)	0.01
In-hospital mortality	11 (9)	2 (2)	0.030

Data are presented as % (*n*) for categorical variables and median (interquartile range) or mean ± standard deviation for continuous variables.

LAAO: left atrial appendage occlusion; RRT: renal replacement therapy; UTI: urinary tract infection; VT/VT: ventricular tachycardiac/ventricular fibrillation.

**Table 3:** Cox proportional hazards analysis

Variable	Univariable			Multivariable		
	HR	95% [CI]	*P*-value	HR	95% [CI]	*P*-value
LAAO	0.29	[0.10–0.80]	0.017	0.39	[0.12–1.23	0.11
Previous sternotomy	2.47	[1.02–5.96]	0.045	1.27	[0.47–3.48]	0.64
Age, years	1.05	[1.01–1.10]	0.027	1.04	[0.99–1.09]	0.088
HTN	1.78	[0.65–4.91]	0.26			
COPD	1.44	[0.42–4.91]	0.56			
DM	2.20	[0.90–5.38]	0.09			
INTERMACS score						
1	1.30	[0.25–6.78]	0.76			
2	1.77	[0.63–4.96]	0.28			
3 (reference)						
4^a^						
Impella	2.08	[0.27–15.80]	0.48			
IABP	1.09	[0.44–2.66]	0.86			
ECMO^b^			0.41			
mPAP	0.99	[0.95–1.03]	0.68			
PCWP	0.99	[0.94–1.04]	0.65			

CI: confidence interval; COPD: chronic obstructive pulmonary disease; DM: diabetes mellitus; ECMO: extracorporeal membrane oxygenation; HR: hazard ratio; HTN: hypertension; IABP: intra-aortic balloon pump; LAAO: left atrial appendage occlusion; mPAP: mean pulmonary artery pressure; PCWP: pulmonary capillary wedge pressure.

a INTERMACS 4 had no mortalities that prevented our using it as a reference level and from putting it into the model as a covariate.

b Mortality difference between patients bridged with ECMO and without determined with the Chi-square test due to there being zero cases of mortality in those bridged with ECMO.

### Thromboembolic events and disabling strokes

**Figure 2:**
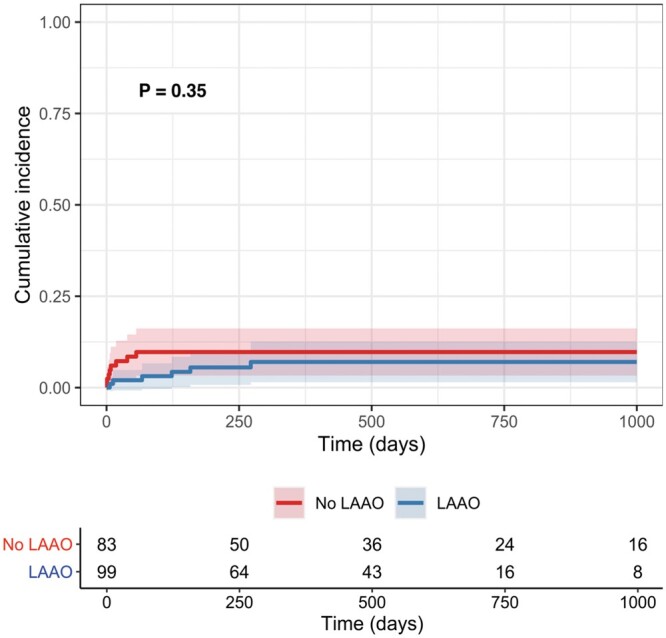
Cumulative incidence curves of thromboembolic events with death and heart transplant as competing events, comparing patients who received left atrial appendage occlusion with HeartMate 3 implant and those who did not. Differences in incidence were assessed via Gray’s test. LAAO: left atrial appendage occlusion.

There were 14 patients who developed TEs, including 13 strokes (7%) and 1 pump thrombosis (0.5%). All 13 strokes, which included patients with both disabling and non-disabling strokes, occurred within the first 6 months following implantation (8 in the no LAAO group vs 5 in the LAAO group, *P* = 0.26). A similar number of patients who did not develop TEs had a previous stroke or transient ischaemic attack as compared to those with TEs (17% vs 14%, *P* = 1.00). Those without TEs also had a similar number of patients with a past medical history of atrial fibrillation compared to those with TEs (50% vs 69%, *P* = 0.25). Complete demographic comparisons between those who developed TEs and those who did not are shown in Table 4. No significant difference in incidence of TEs in each group was found, with the rate of TE occurrence in each <10% at 3 years (Gray’s test: *P* = 0.35; Fig. 2). Regarding disabling strokes specifically, there were zero of such strokes in patients who received LAAO, compared to 6 (7%) in those who did not receive LAAO (*P* = 0.008). Age and previous sternotomy were associated with TEs in the univariable Fine–Gray analysis. LAAO was not associated with TEs in the multivariable Fine–Gray analysis (*P* = 0.10) and no significant risk factors for TEs were identified. Complete Fine–Gray analysis results are shown in Table 5. Two additional supplemental analyses were conducted. The first, in which patients with sternotomy were excluded from the comparison of baseline demographics (Supplementary Material, Table S1), outcomes data (Supplementary Material, Table S2) and cumulative incidence curves comparing TEs in the LAAO versus no LAAO groups (Supplementary Material, Fig. S1) were conducted to account for possible bias in the inclusion of patients with prior sternotomy. A second supplemental analysis comparing outcomes data between patients with non-disabling TEs and disabling TEs was completed assess for predisposing factors to disabling stroke (Supplementary Material, Table S3).

**Table 4:** Baseline demographics and operative characteristics in those who did and did not develop thromboembolic events

Variable	No TEs (*n* = 168)	TEs (*n* = 14)	*P*-value
Baseline demographics			
Age	61 (52–68)	69.44 (56–72.37)	0.053
Sex, male	83 (139)	93 (13)	0.54
HTN	61 (102)	86 (12)	0.12
Stroke/TIA	17 (27)	14 (2)	1.00
PVD	8 (12)	14 (2)	0.33
COPD	12 (20)	0 (0)	0.37
DM	40 (68)	36 (5)	0.78
Afib	50 (82)	69 (9)	0.25
ICM	42 (70)	57 (8)	0.28
Previous sternotomy	24 (40)	50 (7)	0.07
Previous tricuspid surgery	2 (3)	0 (0)	1.00
Previous mitral surgery	7 (11)	7 (1)	1.00
Prior CABG	17 (29)	43 (6)	0.031
BTT	20 (33)	14 (2)	1.00
CHA_2_DS_2_-VASc	3 (2–4)	3.5 (3–4)	0.10
INTERMACS			0.64
1	12 (20)	21 (3)	
2	51 (85)	43 (6)	
3	32 (53)	29 (4)	
4	6 (10)	7 (1)	
IABP	38 (64)	46 (6)	0.57
Impella	2 (4)	14 (2)	0.069
ECMO	7 (11)	17 (2)	0.21
mPAP, mmHg	34.91 ± 10.24	33.5 ± 10.35	0.65
PCWP, mmHg	23 (17–29.5)	19 (15.5–25)	0.60
CVP, mmHg	9 (6–14)	12 (6.75–15.5)	0.42
Fick cardiac output, l/min	3.55 (2.82–4.24)	4.03 (3.27–4.68)	0.10
Preoperative creatinine, mg/dl	1.36 (1.12–1.63)	1.67 (1.43–1.89)	0.10
Preoperative albumin, g/dl	3.8 (3.3–4.1)	4.1 (3.62–4.27)	0.14
Operative characteristics			
CPB time, min	96 (73.5–124.5)	115 (98.25–141.5)	0.055
Concomitant surgery			
Aortic valve surgery	16 (27)	29 (4)	0.26
Mitral valve surgery	16 (27)	0 (0)	0.23
Tricuspid valve surgery	8 (13)	7 (1)	1.00
Intraoperative intracardiac thrombus	3 (5)	0 (0)	1.00
Left ventricle	2	0	
Left atrium	0	0	
Left atrial appendage	1	0	

Data are presented as % (*n*) for categorical variables and median (interquartile range) or mean ± standard deviation for continuous variables.

Afib: atrial fibrillation; BTT: bridge to transplantation; CABG: coronary artery bypass grafting; COPD: chronic obstructive pulmonary disease; CPB: cardiopulmonary bypass; CVP: central venous pressure; DM: diabetes mellitus; ECMO: extracorporeal membrane oxygenation; HTN: hypertension; IABP: intra-aortic balloon pump; ICM: ischaemic cardiomyopathy; mPAP: mean pulmonary artery pressure; PCWP: pulmonary capillary wedge pressure; PVD: peripheral vascular disease; TEs: thromboembolic events; TIA: transient ischemic attack.

**Table 5:** Fine-Gray competing risk regression analysis for thromboembolic events

Variable	Univariable			Multivariable		
	SHR	95% [CI]	*P*-value	SHR	95% [CI]	*P*-value
LAAO	0.61	[0.21–1.74]	0.35	0.41	[0.14–1.20]	0.10
Previous sternotomy	3.11	[1.10–8.77]	0.032	1.78	[0.69–4.59]	0.23
Age, years	1.05	[1.00–1.10]	0.062			
Sex	2.60	[0.33–20.4]	0.36			
HTN	3.61	[0.80–16.20]	0.094			
DM	0.85	[0.29–2.51]	0.76			
PVD	1.88	[0.43–8.28]	0.41			
Afib	2.21	[0.68–7.16]	0.19			
Stroke/TIE	0.80	[0.19–3.41]	0.76			
CHA_2_DS_2_-VASc	1.34	[0.93–1.94]	0.12			
Impella	6.15	[1.35–28]	0.019	1.67	[0.21–13.50]	0.63
IABP	1.35	[0.46–4.01]	0.58			
ECMO	2.83	[0.64–12.5]	0.17			
mPAP	0.99	[0.94–1.04]	0.59			
PCWP	0.99	[0.94–1.05]	0.81			

Afib: atrial fibrillation; CI: confidence interval; DM: diabetes mellitus; ECMO: extracorporeal membrane oxygenation; HTN: hypertension, IABP: intra-aortic balloon pump; LAAO: left atrial appendage occlusion; mPAP: mean pulmonary artery pressure; PCWP: pulmonary capillary wedge pressure; PVD: peripheral vascular disease; SHR: subdistribution hazard ratio; TIE: transient ischaemic event.

## DISCUSSION

This study investigated the effect of LAAO on the development of TEs after HM3 implantation. The rate of TEs was low, with only one pump thrombosis and 13 ischaemic strokes. TE rates did not differ significantly in LAAO versus non-LAAO recipients, and this finding remained true when patients with a previous sternotomy were removed (Supplementary Material, Table S1). However, it cannot be definitively concluded that there was no difference in the rate of TEs between these populations due to the small patient population and overall low number of TEs involved in this study. This is a departure from previous studies, which have demonstrated clear efficacy of LAAO in reducing TEs [9]. The absence of a confirmed difference in TEs between groups may be due to the lack of efficacy of LAAO in preventing TEs after HM3 implant. Were this to be true, a decrease in the efficacy of LAAO in decreasing TEs in HM3 recipients relative to prior generations of LVADs could be resultant from the improved haemocompatibility of the HM3, if the novel device design decreases overall TE risk to such a degree that LAAO provides no added benefit. The primary mechanism underlying pump thrombosis with previous continuous-flow LVADs was thought to be in situ thrombosis along the axial rotors. In the new HM3, the centrifugal design and magnetically levitated component parts are thought to reduce this *in situ* thrombosis that accounted for the majority of pump thromboses with previous continuous-flow LVADs.

Patients with LVADs are known to have the accumulation of thrombus in the left atrium and left atrial appendage despite adequate anticoagulation [13–15]. Thrombus from the left atrium can migrate to the left ventricle and enter the pump as ingested thrombus that can embolize systemically, causing stroke. While this study did not find an association between total ischaemic strokes and transient ischaemic attacks between the LAAO and no LAAO groups, it did show that there were zero disabling strokes in the LAAO group, compared to 6 (7%) in the no LAAO group. Disabling strokes after HM3 implant are more likely resultant from ingested thrombus from the left atrium that the larger gaps between component parts in the HM3 allow to embolize and cause a significant stroke. LAAO may reduce a common source of ingested thrombus that accounts for the lack of disabling strokes seen in patients who received LAAO. All of these disabling strokes occurred in the first 6 months postimplantation, which would be expected to be more related to the HM3 implantation procedure itself and less effected by the improved haemocompatibility of the HM3. In addition, patients’ medical histories affect stroke risk in the first 6 months following implantation. The no LAAO group had a higher-risk profile for perioperative stroke (older age, higher CHA_2_DS_2_-VASc, previous sternotomy). Of the 6 patients with disabling stroke, 3 had bacterial infections in the weeks prior to thrombotic events, indicating that they may have been in a state of hypercoagulability and thus more susceptible to the generation of LA thrombus. The comparison of postoperative outcomes between the non-disabling stroke and disabling stroke groups (Supplementary Material, Table S3) revealed no significant differences in additional postoperative outcome variables. Notably, no patients included in this study had mechanical mitral valves; thus, these were not a source of potential thrombus. LAAO may reduce disabling strokes in these high-risk patients, though due to the higher-risk profile for stroke in the no LAAO group, this study is unable to definitively determine the effect of LAAO on reducing postoperative disabling stroke risk and further study is needed to confirm this finding. In addition, LAAO requires more dissection and procedure time, which must be considered in balancing the possible benefit of LAAO with the increased operative risk associated with its use.

Patients with HM3 who develop bleeding following implant must be carefully monitored and receive anticoagulation based on their symptoms. However, there are little data on the safety of withholding anticoagulation in HM3 patients with regard to TE risk. While this study found no difference in the overall incidence of TEs between those receiving LAAO and those not receiving LAAO at the time of HM3 implantation, LAAO has been shown to decrease anticoagulation requirements and stroke risk in patients with atrial fibrillation [16–18]. LAAO may give clinicians more confidence in withholding anticoagulation for an HM3 patient who develops bleeding, though further studies are required to determine the safety of withholding anticoagulation in HM3 patients and the role of LAAO in reducing TE risk.

### Limitations

Due to resternotomy patients not generally being candidates for LAAO, there is a source of bias in this study’s data. However, a large proportion of the cohort had a prior sternotomy and it was deemed most clinically useful to include these patients in analyses as many patients undergoing HM3 implant have had a prior sternotomy. This potential source of bias was accounted for by including previous sternotomy as a cofactor in the multivariable Fine and Gray analysis. For the interest of the reader, an analysis with previous sternotomy patients excluded has been provided, which includes a comparison of baseline demographics (Supplementary Material, Table S1), outcomes data (Supplementary Material, Table S2) and cumulative incidence curves comparing TEs in those who did and did not receive LAAO (Supplemental Material, Fig. S1). The incidence of TEs did not differ significantly between groups (Gray’s test: *P* = 0.39).

The retrospective, uncontrolled nature of this study creates additional limitations. First, the LAAO and no LAAO groups are not similar given differences in baseline characteristics that are representative of the widespread etiologies of heart failure (Table 1), and propensity score matching failed to adequately match patients because of these differences. This study is also limited in that LAAOs were done at the surgeon’s discretion and without a standardized protocol for determining specific patients for whom it is indicated, which may lead to bias in the study. Lastly, the retrospective design of this study prevents accounting for all possible confounding variables that prevented the determination of any causal relationships. Due to this being a single-centre study, outcomes may not be applicable to other centres and multicentre studies are needed to confirm these findings. With a very small sample size, this study may lack the statistical power to determine an association between LAAO and TE prevention after HM3 implantation, if present.

## CONCLUSIONS

A low number of TEs was observed in HM3 recipients. No statistically significant difference in the rate of TEs between no LAAO and LAAO groups was found in this patient population. While it cannot be definitively concluded that LAAO did not further reduce the overall rate of TEs in this patient population, its use may be beneficial in preventing disabling ischaemic strokes after HM3 implantation. LAAO’s potential role in preventing disabling strokes presents an opportunity for the future study.

## SUPPLEMENTARY MATERIAL

Supplementary material is available at *ICVTS* online.

## Funding

No funding was received for this study.

**Conflict of interest:** Paolo C. Colombo, Gabriel Sayer and Yoshifumi Naka have received consulting fees from Abbott. Nir Uriel has received grant support and consulting fees from Abbott and Medtronic. The remaining authors have no conflicts of interest to disclose.

##  

### Author Contributions

**Andrew Melehy:** Conceptualization; Data curation; Formal analysis; Investigation; Methodology; Writing—original draft; Writing—review & editing. **Gillian O'Connell:** Data curation; Methodology; Writing—review & editing. Yuming Ning: Formal analysis; Methodology; Writing—review & editing. **Paul Kurlansky:** Formal analysis; Methodology; Writing—review & editing. **Yuji Kaku:** Conceptualization; Writing—review & editing. **Veli Topkara:** Conceptualization; Writing—review & editing. **Melana Yuzefpolskaya:** Conceptualization; Writing—review & editing. **Paolo C. Colombo:** Conceptualization; Writing—review & editing. **Gabriel Sayer:** Conceptualization; Writing—review & editing. **Nir Uriel:** Conceptualization; Writing—review & editing. **Yoshifumi Naka:** Conceptualization; Project administration. **Koji Takeda:** Conceptualization; Investigation; Project administration; Supervision; Writing—original draft; Writing—review & editing.

### Reviewer Information

Interactive CardioVascular and Thoracic Surgery thanks Luca Bertoglio, Roberto Lorusso, Massimiliano Meineri and the other, anonymous reviewer(s) for their contribution to the peer review process of this article.

## Supplementary Material

ivab285_Supplementary_Data

## Notes

Presented at the Meeting of the International Society for Heart and Lung Transplantation, Virtual, 24–28 April 2021.

## Abbreviations

**ABBREVIATIONS**
HM3: HeartMate 3
LAAO: Left atrial appendage occlusion
LVADs: Left ventricular assist devices
TEs: Thromboembolic events
