# Supplementary material for: Role of left atrial appendage occlusion in patients with HeartMate 3
Source: Interact Cardiovasc Thorac Surg. 2021 Oct 18;34(4):668–75. doi: 10.1093/icvts/ivab285 (PMC8972327; doi:10.1093/icvts/ivab285)
Supplement: ivab285_Supplementary_Data [file ivab285_supplementary_data.zip › LAAO_supplemental_062521.docx]

| **Supplemental Table 1:** Baseline demographics and operative characteristics comparing patients who did and did not receive LAAO, excluding those with a previous sternotomy | | | | |
| --- | --- | --- | --- | --- |
| variable | | no LAAO (n= 40) | LAAO (n= 95) | P-value |
| **Baseline Demographics** |  |  |  |  |
| Age, y | 62 (52-68) | 59 (51-63) | 0.17 |  |
| Sex, male | | 85% (34) | 79% (75) | 0.57 |
| HTN | | 57% (23) | 61% (58) | 0.85 |
| Stroke/ TIA | | 2% (1) | 5% (5) | 0.67 |
| PVD | | 5% (2) | 5% (4) | 1.00 |
| COPD | | 8% (3) | 13% (12) | 0.55 |
| DM | | 38% (15) | 35% (33) | 0.91 |
| Afib | | 48% (19) | 47% (43) | 1.00 |
| ICM | | 35% (14) | 26% (25) | 0.42 |
| Previous Mitral Surgery | | 5% (2) | 2% (2) | 0.59 |
| Prior CABG | | 2% (1) | 0% (0) | 0.3 |
| BTT | | 30% (12) | 15% (14) | 0.07 |
| CHA₂DS₂-VASc | | 3 (2-4) | 3 (2-4) | 0.56 |
| INTERMACS | |  |  | 0.63 |
| 1 | | 18% (7) | 11% (10) |  |
| 2 | | 28% (11) | 32% (30) |  |
| 3 | | 2% (1) | 5% (5) |  |
| 4 | | 52% (21) | 53% (50) |  |
| IABP | | 32% (13) | 45% (42) | 0.24 |
| Impella | | 5% (2) | 1% (1) | 0.21 |
| ECMO | | 10% (4) | 8% (7) | 0.73 |
| mPAP, mmHg | | 37.10 ± 9.69 | 35.41 ± 9.96 | 0.37 |
| PCWP, mmHg | | 25.50 ± 8.95 | 23.74 ± 9.27 | 0.30 |
| CVP, mmHg | | 11 (8-14) | 9 (6-15) | 0.25 |
| Fick cardiac output, L/min | | 3.49 (2.98-4.04) | 3.56 (2.82-4.19) | 0.70 |
| Preoperative creatinine, mg/dL | | 1.49 ± 0.48 | 1.39 ± 0.41 | 0.30 |
| Preoperative albumin, g/dL | | 3.60 (3.20-4.10) | 3.80 (3.40-4.20) | 0.20 |
| **Operative Characteristics** | |  |  |  |
| CPB Time, min | | 86 (61-111) | 95 (72-131) | 0.25 |
| Concomitant Surgery | |  |  |  |
| Aortic Valve Surgery | | 10% (4) | 16% (15) | 0.43 |
| Mitral Valve Surgery | | 5% (2) | 19% (18) | 0.06 |
| Tricuspid Valve Surgery | | 8% (3) | 6% (6) | 0.72 |
| Intraoperative Intracardiac Thrombus | | 5% (2) | 3% (3) | 0.63 |
|  | | Left Ventricle 2 | 2 |  |
|  | | Left Atrium 0 | 0 |  |
|  | | Left Atrial Appendage 0 | 1 |  |
| Data presented as % (n) for categorical variables and median (interquartile range) or mean ± standard deviation for continuous variables.  Afib, atrial fibrillation; BTT, bridge to transplantation; CABG, coronary artery bypass grafting; COPD, chronic obstructive pulmonary disease; CPB, cardiopulmonary bypass; DM, diabetes mellitus; ECMO, extracorporeal membrane oxygenation; HTN, hypertension; IABP, intra-aortic balloon pump; ICM, ischemic cardiomyopathy; LAAO, left atrial appendage occlusion; mPAP, mean pulmonary artery pressure; PCWP, pulmonary capillary wedge pressure | | | | |

| **Supplemental Table 2:** Outcomes Data comparing patients who did and did not receive LAAO, excluding those with a previous sternotomy | | | |
| --- | --- | --- | --- |
| variable | no LAAO (n= 83) | LAAO (n= 99) | P-value |
| Postoperative atrial fibrillation | 40% (16) | 39% (37) | 1.00 |
| Postoperative VT/VF | 18% (7) | 23% (22) | 0.50 |
| Postoperative sepsis | 13% (5) | 19% (18) | 0.46 |
| Postoperative UTI | 10% (4) | 21% (20) | 0.15 |
| Postoperative takeback | 10% (4) | 18% (17) | 0.31 |
| Postoperative RRT | 8% (3) | 4% (4) | 0.42 |
| Postoperative tracheostomy | 5% (2) | 9% (9) | 0.51 |
| Thromboembolic events | 2% (1) | 6% (6) | 0.67 |
| Ischemic strokes | 2% (1) | 5% (5) | 0.67 |
| Disabling strokes | 2% (1) | 0% (0) | 0.30 |
| Cardiac transplants | 32% (13) | 19% (18) | 0.14 |
| Hospital stay, d | 27 (20-46) | 27 (21-36) | 0.71 |
| Overall mortality | 15% (6) | 5% (5) | 0.08 |
| In hospital mortality | 8% (3) | 2% (2) | 0.15 |
| Data presented as % (n) for categorical variables and median (interquartile range) or mean ± standard deviation for continuous variables.  LAAO, left atrial appendage occlusion; RRT, renal replacement therapy; UTI, urinary tract infection; VT/VF, ventricular tachycardiac/ ventricular fibrillation | | | |

| **Supplemental Table 3:** Outcomes Data comparing patients who did and did not have disabling stroke | | | |
| --- | --- | --- | --- |
| variable | no disabling stroke (n= 176) | Disabling stroke (n= 6) | P-value |
| Postoperative atrial fibrillation | 39% (68) | 33% (2) | 1.00 |
| Postoperative VT/VF | 21% (37) | 17% (1) | 1.00 |
| Postoperative sepsis | 17% (30) | 33% (2) | 0.29 |
| Postoperative UTI | 18% (32) | 17% (1) | 1.00 |
| Postoperative takeback | 15% (27) | 0% (0) | 0.59 |
| Postoperative RRT | 8% (14) | 0% (0) | 1.00 |
| Postoperative tracheostomy | 9% (16) | 20% (1) | 0.39 |
| Thromboembolic events | 5% (8) | 100% (6) | < 0.01 |
| Ischemic strokes | 4% (7) | 100% (6) | < 0.01 |
| LAAO | 56% (99) | 0% (0) | 0.01 |
| Cardiac transplants | 21% (37) | 17% (1) | 1.00 |
| Overall mortality | 9% (16) | 67% (4) | 0.01 |
| In hospital mortality | 4% (7) | 67% (4) | < 0.01 |
| Data presented as % (n) for categorical variables and median (interquartile range) or mean ± standard deviation for continuous variables.  LAAO, left atrial appendage occlusion; RRT, renal replacement therapy; UTI, urinary tract infection; VT/VF, ventricular tachycardiac/ ventricular fibrillation | | | |

Supplemental Figure 1


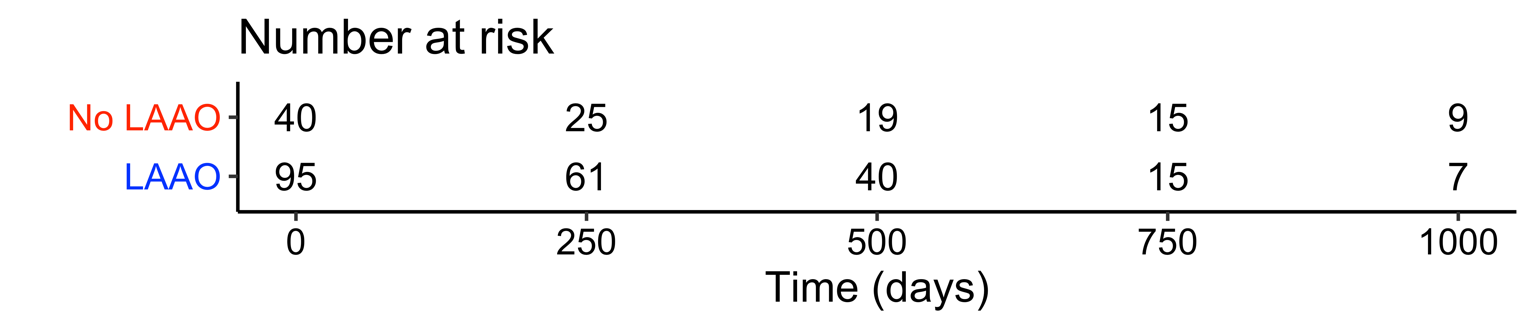

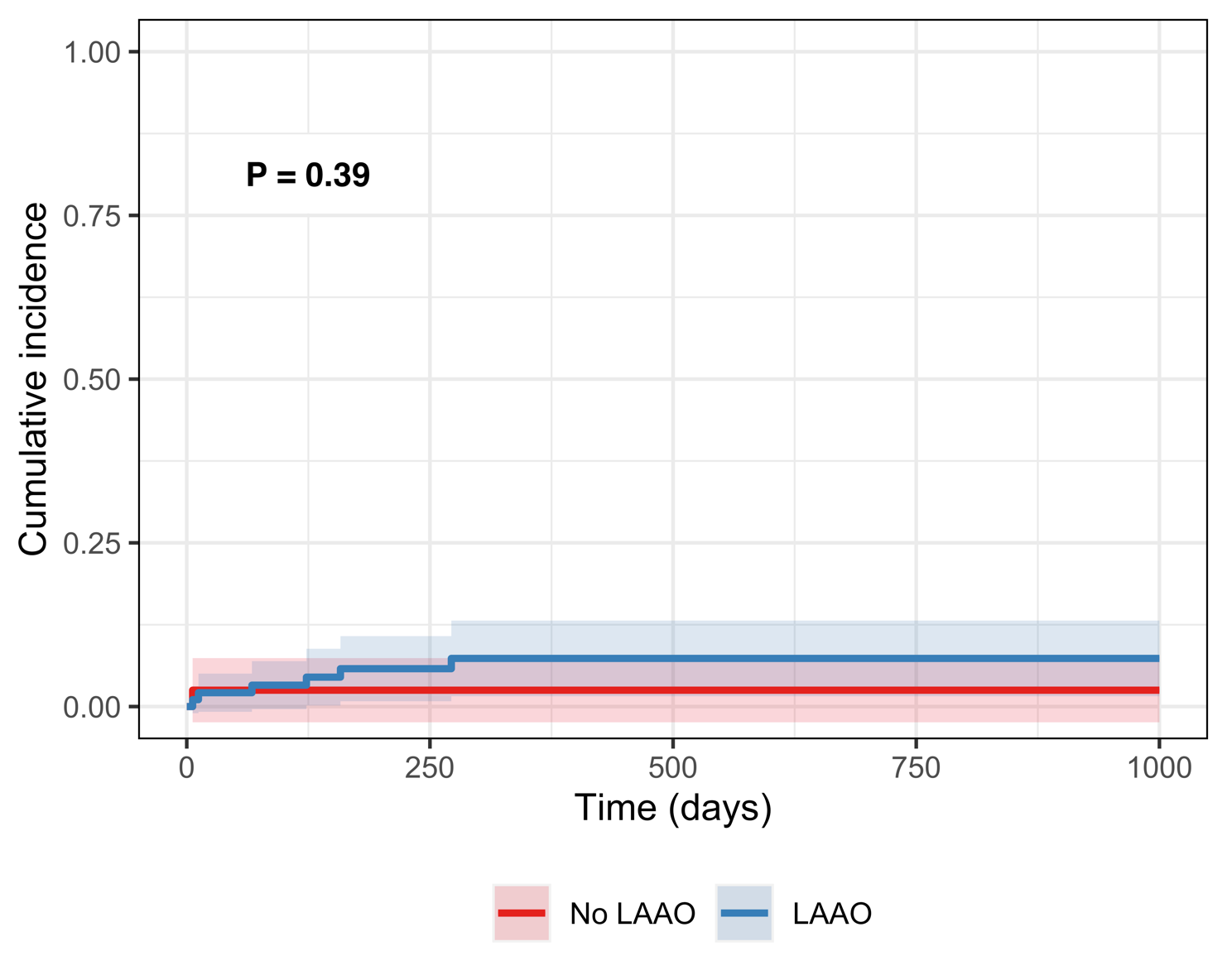


**Supplemental Figure 1.** Cumulative incidence curves of TEs with death and heart transplant as competing events, comparing patients who received LAAO with HM3 implant and those who did not, excluding those with a previous sternotomy. Differences in incidence were assessed via Gray’s test. TE, thromboembolic event, LAAO, left atrial appendage occlusion, HM3, HeartMate 3.
