# Supplementary figures and images for: Role of left atrial appendage occlusion in patients with HeartMate 3
Source: Interact Cardiovasc Thorac Surg. 2021 Oct 18;34(4):668–75. doi: 10.1093/icvts/ivab285 (PMC8972327; doi:10.1093/icvts/ivab285)

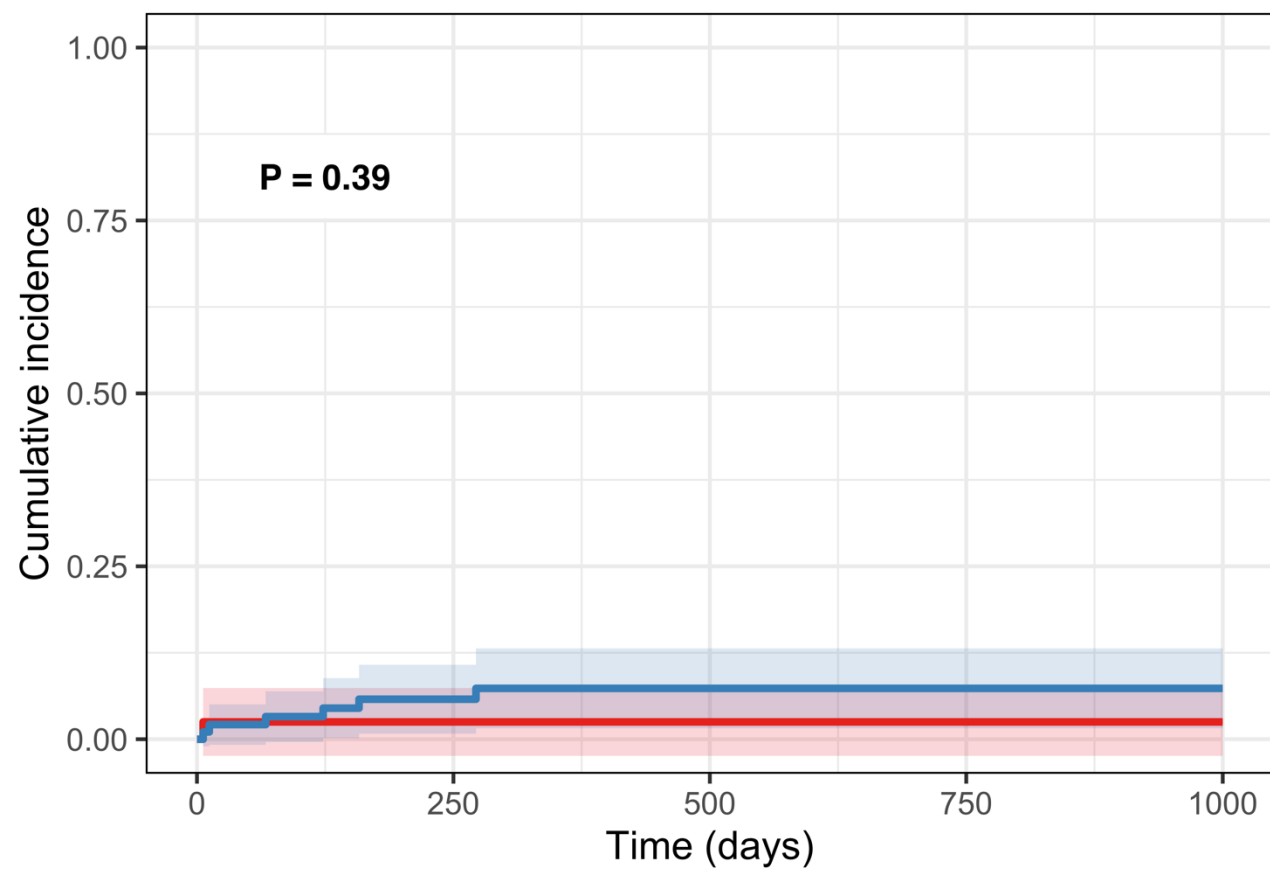

|         |                                                   |     |     |     |      |
|---------|---------------------------------------------------|-----|-----|-----|------|
|         | <div> <div></div> No LAAO <div></div> LAAO </div> |     |     |     |      |
| No LAAO | 40                                                | 25  | 19  | 15  | 9    |
| LAAO    | 95                                                | 61  | 40  | 15  | 7    |
|         | 0                                                 | 250 | 500 | 750 | 1000 |
|         | Time (days)                                       |     |     |     |      |

Supplement: ivab285_Supplementary_Data [file ivab285_supplementary_data.zip › LAAO_Supp figure_0921.pdf]
